# ABO Incompatible Liver Transplantation as a Bridge to Treat HELLP Syndrome

**DOI:** 10.1155/2009/713937

**Published:** 2009-09-28

**Authors:** Kathleen Connor, Raymond A. Rubin, Roshan Shrestha, Mark Johnson, Marty Sellers, Brad Butler

**Affiliations:** ^1^Department of Liver Transplantation, Piedmont Hospital, Atlanta, GA 30309, USA; ^2^Liver Center of Atlanta, Piedmont Hospital, Atlanta, GA 30309, USA; ^3^Department of Pathology, Piedmont Hospital, Atlanta, GA 30309, USA

## Abstract

The following is a case report of a primiparous woman who developed fulminant liver failure in the setting of HELLP syndrome complicated by hepatic rupture. It is unique in that a timely ABO compatible liver donor was unavailable, necessitating the transplantation of an ABO incompatible organ. Despite aggressive therapy, *severe reperfusion injury* and humoral rejection dictated retransplantation with an ABO compatible organ *on postoperative day 15*, resulting in rapid clinical recovery.

## 1. Introduction

Hemolysis, elevated liver enzymes, and low platelet count (HELLP) syndrome is an uncommon, but potentially life threatening cause of acute liver failure in peripartum women. It occurs in 0.2–0.6% of all pregnancies, but can be present in up to 4–12% of cases with severe pre-eclampsia [1]. In about 30% of cases, the disease presents postpartum, usually within 48 hours of delivery [2]. HELLP syndrome is associated with a mortality of 1.1% and management typically involves urgent delivery and aggressive supportive care. The mortality associated with this condition is dramatically worsened when there is hepatic rupture [3, 4]. The following is a report of *such a case initially treated with ABO incompatible liver transplantation and ultimately with ABO compatible transplantation*.

## 2. Case Report

A 28-year-old Gravida 1 Caucasian female without significant medical history presented to her obstetrician for a routine office visit at 37 weeks gestation. Her examination revealed a blood pressure of 150/98 mmHg, 1+ pedal edema, and 1+ proteinuria. Further laboratories demonstrated a hemoglobin of 10.1 g/dL, platelet count 106 K/mm^3^, aspartate aminotransferase (AST) 290 IU/L, and lactate dehydrogenase (LDH) of 371 IU/L. She was presumptively diagnosed with HELLP syndrome and admitted for urgent Caesarean section.

While the surgery was uneventful, several hours later she developed acute lower back pain and hypotension. Repeat laboratory studies showed hemoglobin of 6.0 g/dL, platelet count 10 K/mm^3^, AST 1696 IU/L, alanine aminotransferase (ALT) 1253 IU/L, LDH 1262 IU/L. Abdominal ultrasound and CT demonstrated subcapsular and perihepatic hemorrhage with free intraabdominal blood. She was transfused red blood cells and platelets and treated with high doses of intravenous corticosteroids. Vasopressor agents were required to maintain adequate blood pressure and continuous renal replacement therapy (CRRT) was instituted for anuric renal failure.

Four days after delivery, her mental status deteriorated. She was endotracheally intubated and transferred to our institution for further management. Upon arrival, laboratory studies showed hemoglobin 8.4 g/dL, platelets 39 K/mm^3^, international normalized ratio 1.49, factor V 32%, blood type O, AST 1319 IU/L, ALT 763 IU/L, bilirubin 7.6 mg/dL, LDH 2215 IU/L, creatinine 4.1 mg/dL, and lactic acid 5.1 mmol/L. Serologic evaluation for other causes of liver disease and human immunodeficiency virus was negative. CMV IgM was negative and IgG was positive. There was no evidence of abdominal compartment syndrome. Head CT showed no apparent cerebral edema.

As there were no signs of improvement despite intensive therapy for fulminant hepatic failure, six days postpartum she was listed for liver transplantation as a United Network of Organ Sharing (UNOS) status 1. Over the next five days, her clinical course deteriorated further, necessitating 100% forced inspiratory oxygen and high dose vasopressors. Since no suitable ABO compatible livers had been offered, on the fifth day after listing (11 days postpartum), the decision was made to accept a blood type A1 liver.

**Figure 1:**
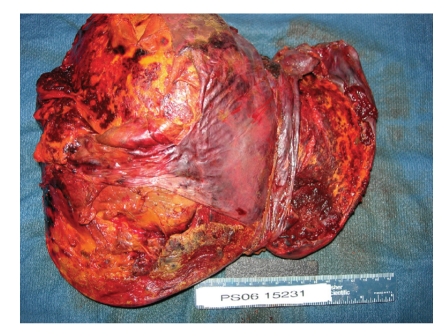
Native liver: 3000 g liver (expected weight 1500–1800 g) with dull yellow-green geographic necrosis and subcapsular blood.

**Figure 2:**
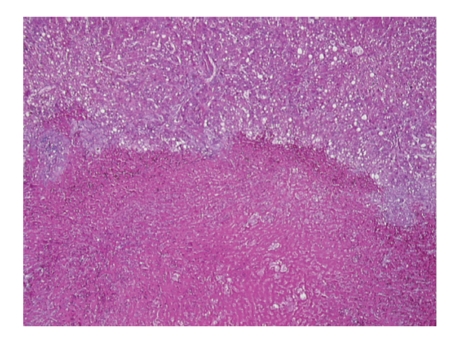
Native liver (40x, H&E section): at low power, one may appreciate the interface between viable and necrotic hepatic tissue.

**Figure 3:**
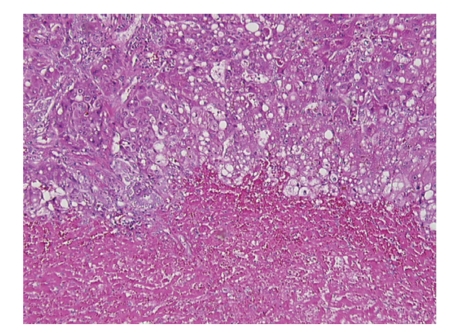
Native liver (100x, H&E section): higher power view of the interface in the native liver between viable and necrotic hepatic tissue. Note the cytologic changes of hepatocellular injury in the viable tissue.

Orthotopic liver transplantation was performed using the piggyback technique. The donor was 31 years old with a body mass index of 24 kg/m^2^. No gross fatty change was appreciated; liver biopsy was not performed. Cold ischemic time was six hours and 40 minutes and warm ischemic time was 23 minutes. Gross inspection of the native liver revealed obvious hepatic rupture, with the organ surrounded by hematoma (Figure 1). Histology demonstrated massive hepatic necrosis, marked steatosis, widespread bile ductular proliferation, profound cholestasis, and necrotic vessels with intravascular thrombi (Figures 2 and 3).

**Figure 4:**
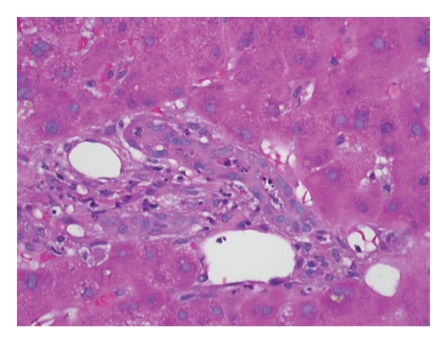
First allograft (400x, H&E section): portal areas show areas of cholangitis. No features of acute cellular rejection. The parenchyma surrrounding this portal area appears viable.

She was treated with a renal-sparing immunosuppressive regimen including *high dose intravenous immune globulin*, thymoglobulin, methylprednisolone, mycophenolate Mofetil, and, beginning on day 7, tacrolimus. Daily plasma exchange was performed with blood group A antigen specific immunoadsorbent columns. Over the first seven days, serum transaminases and coagulation studies steadily improved, although she continued to require full ventilatory support and CRRT. Although somnolent, she became arousable to stimulation. Over the next 48 hours, however, the transaminases and bilirubin worsened**. **Liver biopsy demonstrated mild reperfusion injury, mild cholangitis, spotty apoptosis, but no cytopathic effect or endothelialitis. A C4d stain was negative. *Direct antiglobulin and Coomb's tests were negative on three separate occasions*. Biliary obstruction, vascular thromboses and infections, including cytomegalovirus, were excluded (Figure 4). On postoperative day 11, she was comatose and laboratory studies showed bilirubin 14.5 mg/dL, AST 1747 IU/L, ALT 939 IU/L, INR 1.75. As a result of continued deterioration, she was relisted for transplantation with a MELD score of 35 on postoperative day 15.

**Figure 5:**
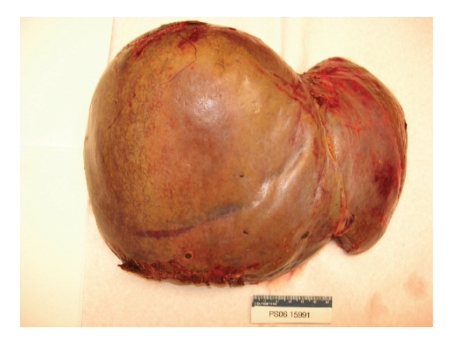
Explant of Allograft: 2531 g liver (expected weight 1500-1800 g) congested parenchyma with approximately 30% necrosis of the left lobe.

**Figure 6:**
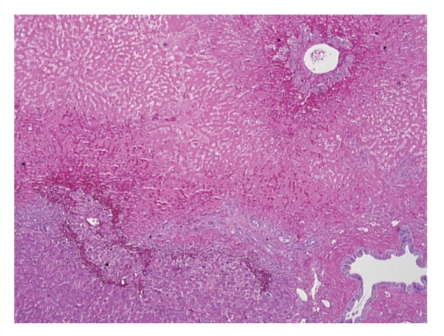
Allograft (40x, H&E section).

**Figure 7:**
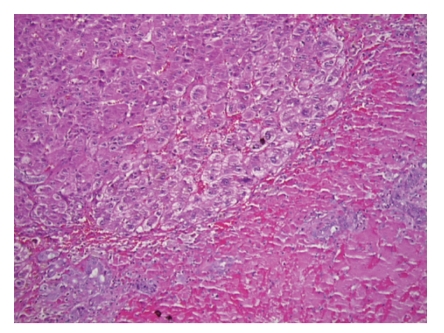
Allograft (100x, H&E section): higher power view of large zones of necrosis abutting adjacent viable hepatic tissue.

An ABO compatible liver became available within 24 hours. Histology of the failed allograft demonstrated numerous areas of hemorrhagic necrosis; 30% of the left lobe was completely necrotic (Figures 5, 6, and 7). On post-operative day 1, the patient became alert and oriented. Over the next several weeks she was extubated and CRRT was discontinued. She was discharged on post-operative day 32 (17 days after re-transplantation) with baseline mental status and normal serum liver tests and creatinine. She and her baby are alive and well thirty months after transplant.

## 3. Discussion

Hepatic rupture is an unusual, but potentially grave, complication of HELLP syndrome. Emergency laparotomy is required for acute compartment syndrome or ongoing bleeding. Interventions at the time of surgery can include temporary packing of the liver, evacuation of a hematoma, suturing of a laceration, ligation of the hepatic artery, and/or resection of necrotic liver [5, 6].

Liver transplantation is rarely required for HELLP syndrome, but is more likely to be necessary if there is hepatic rupture [7]. Transplantation may be necessary for unrelenting bleeding or fulminant liver failure. Shames et al. detailed such a case and found only 7 other patients in the UNOS database from 1987–2004 in whom HELLP syndrome was the indication for liver transplantation. Six of these seven patients survived and two required re-transplantation, both for infectious issues [5].

Our case report is unique in that *an ABO incompatible liver (A1 → O) unintentionally served as a “bridge” to successful transplantation with an ABO compatible organ to treat fulminant hepatic failure secondary to HELLP syndrome*. Once the decision was made to list our patient as a UNOS status 1, no suitable blood type O liver was available for five days, two days longer than the median wait time in our region. *Given the rapidly declining clinical status of a young and otherwise healthy new mother*, it was determined that the potential benefit of crossing the ABO barrier outweighed the medical and ethical risks. *We proceeded with ABO incompatible transplantation with full expectations of patient and graft survival. Only when the ABO incompatible graft failed from a combination of reperfusion injury and humoral rejection, did we resort to relisting the patient for an ABO compatible organ*.

ABO incompatible liver transplantation is infrequently utilized since the high risks of antibody mediated rejection, vascular thrombosis, and ischemic bile duct complications are associated with reduced patient and graft survival [8–11]. Transplanting A1 (rather than A2) → O presents greater challenges as enhanced expression of A antigen may intensify isohemagglutinin mediated rejection.

Several strategies have been employed to try to minimize humoral rejection in ABO incompatible liver transplantations. Pre- and posthigh volume plasma exchange or blood group specific immunoadsorbent columns may be utilized to reduce preformed complement-fixing antibodies [12–14]. Some centers follow hemagglutinin titers to monitor such therapy, particularly during the high risk period of the first few postoperative weeks [12, 13]. While older aggressive immunosuppressive regimens complemented cyclophosphamide and OKT3 treatment with splenectomy [12], more recently, mycophenolate mofetil, daclizumab induction, and/or anti-CD20 monoclonal antibody have been used successfully in small case series [14]. In Japan, where up to 5% of adult living donor liver transplantation involve ABO incompatible organs, supplemental postoperative portal vein infusions with methylprednisolone, prostaglandin E1, and/or gabexate mesylate have been used to control local disseminated intravascular coagulation [15].

In our case, aggressive immunosuppression, *intravenous immune globulin*, and immunoadsorption with immobilized A antigen was associated with fair early graft function. The clinical deterioration which occurred after approximately one week, combined with the biopsy findings of hepatic necrosis, cholangitis, and apoptosis was consistent with refractory antibody-mediated rejection. Definitively diagnosing humoral rejection as the sole cause of graft failure may be challenging. Biopsy findings are typically not path gnomonic, although periportal edema and necrosis may be early findings [16]. Other coexisting pathology, such as preservation injury and acute cellular rejection, may occur simultaneously and confound biopsy interpretation [17]. Sinusoidal deposition of hemagglutinins and their complements may be seen by immunohistochemical analysis in humoral rejection [16]. While not confirmatory in our case, positive C4d (a degraded product of complement C4) immunostaining has been associated in some instances with high antidonor antibody titers and relatively poorer overall survival rate [17]. With our patient, one could speculate whether further intensifying the immunosuppression or performing splenectomy would have stabilized the clinical course or merely increased the likelihood of sepsis and *multiorgan failure*. The rapid clinical improvement once an ABO compatible liver was transplanted further supports the hypothesis that antibody mediated rejection *contributed to* failure of the first graft.

Patients with HELLP syndrome complicated by hepatic rupture should be transferred to a liver transplantation center for management and emergent evaluation for liver transplantation. While ABO incompatible transplantation should be considered in only exceptional circumstances, antigen-specific immunoadsorption, aggressive immunosuppression, consideration of portal vein infusion therapy, and even splenectomy may optimize outcomes in this setting. Even utilizing these extraordinary measures, graft and patient survival still remains inferior to results with ABO compatible livers.
